# Supplementary material for: Morphometric frequency and spectrum of gamma-ray-induced chlorophyll mutants identified by phenotype and development of novel variants in lentil (Lens culinaris Medik.)
Source: PLoS One. 2023 Jun 12;18(6):e0286975. doi: 10.1371/journal.pone.0286975 (PMC10259775; doi:10.1371/journal.pone.0286975)
Supplement: Attachment — Submitted filename: Lentil mutation research paper [PLOS ONE] revise.docx [file pone.0286975.s001.docx]

**Morphometric frequency and spectrum of gamma-ray-induced chlorophyll mutants identified by phenotype and development of novel variants in lentil (*Lens culinaris* Medik.)**

Biswajit Pramanik^1^, Sandip Debnath^1*^, Mehdi Rahimi^2^, Md Mostofa Uddin Helal^3^, Rakibul Hasan^4*^

^1^Department of Genetics and Plant Breeding, Palli Siksha Bhavana (Institute of Agriculture),

Visva-Bharati University, Sriniketan, West Bengal, India.

^2^Department of Biotechnology, Institute of Science and High Technology and Environmental

Sciences, Graduate University of Advanced Technology, Kerman, Iran.

^3^Institute of Wheat Research, State Key Laboratory of Sustainable Dryland Agriculture, Shanxi Agricultural University, Linfen, 041000, China.

^4^Department of Plant Pathology and Seed Science, Sylhet Agricultural University, Sylhet -3100, Bangladesh

*Corresponding author: [rakib.ppath@sau.ac.bd](mailto:rakib.ppath@sau.ac.bd) and [sandip.debnath@visva-bharati.ac.in](mailto:sandip.debnath@visva-bharati.ac.in)

**Abstract**

Genetic variations are a crucial source of germplasm heterogeneity because they contribute to the development of new traits for plant breeding by offering an allele resource. Gamma rays have been widely used as physical agents to produce mutations in plants, and their mutagenic effect has attracted much attention. Nonetheless, there are few studies on the whole mutation spectrum in large-scale phenotypic evaluations. To comprehensively examine the mutagenic effects of gamma irradiation on lentils, biological consequences on the M_1_ generation and substantial phenotypic screening on the M_2_ generation were undertaken. The purpose of this study was to induce unique genetic variability in lentils by irradiating seeds of the variety *Moitree* with a range of acute gamma irradiation doses (0, 100, 150, 200, 250, 300, and 350 Gy). Additionally, this research concentrated on finding the GR_50_ value while taking seedling parameters into account and on examining the status of pollen fertility while comparing the effects of the gamma irradiation dosages stated previously. The GR_50_ value was determined to be 217.2 Gy using the seedling parameters. On the other hand, pollens from untreated seed-grown plants were almost 85 percent fertile, but those treated with the maximum dosage (350 Gy) were approximately 28 percent fertile. Additionally, the decrease in pollen fertility and the increasing dosages of radiation corroborated the detrimental impact of gamma rays on seedling height. Additionally, numerous chlorophyll and morphological mutants were produced in the subsequent generation, with the 300 Gy-treated seeds being the most abundant, followed by the 250 Gy-treated seeds. This demonstrated that an appropriate dosage of gamma rays was advantageous when seeking to generate elite germplasm resources for one or a number of traits. These investigations will contribute to a comprehensive understanding of the mutagenic effects and actions of gamma rays, which will provide a basis for the selection and design of suitable mutagens. Hence, it will facilitate the development of more controlled mutagenesis protocols for plant breeding.

**Keywords:** Gamma irradiation; Genetic variation; GR_50_; Lentil; Mutation breeding; Pollen fertility

1. Introduction

Lentil (*Lens culinaris* Medik.) is an important pulse crop in India. It is a member of the Fabaceae family with chromosome number 2n = 2x = 14 [1]. It is an annual edible legume with lens-shaped pods and purse-shaped seeds [2]. This oldest known legume originated in the Near East [3], from whence it spread to Canada, Turkey, and Southeast Asian nations such as India, China, Nepal, Afghanistan, Pakistan, and Bangladesh, among others [4]. Lentil seeds are a good source of a variety of proteins, vitamins, and minerals that are required for human nutrition [5]. Additionally, the availability of more tryptophan and lysine makes it a good complement to rice or wheat, which is deficient in that specific amino acid and thus offers balanced nutrition in the human diet. Because the legume "lentil" is a high-protein legume (containing approximately 24.1% protein), crop improvement techniques may have a major impact [6]. Surprisingly, socioeconomic and agroecological constraints are the primary impediments to the development of this food legume across India, despite its great nutritional value. Numerous biotic and abiotic stress conditions significantly reduce the yield potential of lentils, particularly in resource-poor areas worldwide [7]. The indigenous cultivars of lentil in India belonged to a specific ecotype (Pilosae), which appears to lack notable variability for phenological, morphological, yield- and resistance-associated traits, resulting in the failure of lentil improvement programmes for a longer duration [8]. The day length bottleneck hampered the transfer of lentil germplasm into the Indo-Gangetic plain, resulting in decreased yield potential in that particular geographical region [9]. Additionally, the small and delicate blooms hampered the success rate of artificial hybridization by up to 20%–50% due to the difficulties of emasculation and pollination, which resulted in injury during these procedures. As a consequence, cross-pollination has become a time-consuming process in lentils [8]. Additionally, the genus *Lens* was notable for the development of crossability barriers during interspecific hybridization within and across species [10]. Eventually, these processes collectively resulted in the lentil's narrow genetic base. Various scholars previously identified this narrow genetic basis as the primary restraint on lentil production [11]. Additionally, adaptive specificity and repeated failures to employ various unproductive foreign germplasms contribute to the failure of lentil genetic progress [12]. Thus, given the parameters outlined above, mutagenesis might be seen as a supplementary and novel breeding strategy. It is capable of generating such a variation that does not exist in the organism's genetic background. As a result, this feature may be used to enhance a plethora of desirable characteristics in a plant species. A wide range of mutation methods have been used, including physical and chemical mutagens such as gamma radiation, X-rays, heavy metal ions, and protons, as well as biotechniques such as genetic modification, transgenic, or gene editing based on CRISPR/Cas9 technology. Many different forms of DNA damage may be caused by photon radiation, including the nucleotide alterations and strand breaks caused by oxidized bases, abasic sites, single-strand breaks, and double-strand breaks that gamma radiation can cause. If this DNA damage is not addressed or is repaired ambiguously, mutations, such as single-base substitutions, deletions, insertions, inversions, or translocations, may arise at the genome scale and eventually lead to alterations in the phenotypic traits of the organisms [13]. Numerous researchers have proposed and implemented mutation breeding as an effective tool for this kind of improvement in a variety of crops [11, 14-22]. Amin, et al. [11] published a report indicating a dose-dependent decrease in the germination of lentil seeds treated with MMS (methyl methanesulfonate). Additionally, they noted that 2% (v/v) DMSO (dimethyl sulfoxide) inhibited MMS action. Gaur, et al. [17] have observed similar results utilizing gamma rays as a mutagen on pigeon pea. Khursheed, et al. [18] evaluated the combined impact of gamma rays and ethyl methanesulfonate (EMS) on the mutagenesis potential of faba bean (*Vicia faba* L.) seeds by determining the germination percentage and seedling height.

As a result, this research was undertaken to find the optimal dosage of acute gamma-irradiated mutagenesis for the improvement of lentil agronomically significant features to achieve a greater mutation frequency [22, 23]. Additionally, the immediate impact of gamma irradiation on seedling and sterility parameters is discussed in this section. Additionally, the influence of determining the GR_50_ value by the detection of a large number of mutants, both chlorophyll and morphological, was shown in this study. These studies focused on the lentil variety "Moitree." It is a well-known variety among farmers in West Bengal, India, as well as a national check because of its considerably greater production performance than other varieties, even when seeded late. As a result, a group of researchers used this variety extensively in their experiments [24-26]. The creation of numerous chlorophyll and morphological mutants in the subsequent generation would be crucial for the identification of new alleles and might potentially be released as varieties or prebreeding material in the near term if they are found to be economically sustainable and agronomically useful.

2. Materials and Methods

*2.1. Determination of GR_50_ value*

Approximately 35000 homogenous, uniform, dry and healthy seeds of lentil (variety: *Moitree*) were collected from the Department of Genetics and Plant Breeding, Palli Siksha Bhavana (Institute of Agriculture), Visva-Bharati, Sriniketan. Among them, every 1/7^th^ of the total seeds, i.e., almost 5000 seeds were exposed to acute gamma irradiation of seven doses including 0, 100, 150, 200, 250, 300, and 350 Gy, respectively, at the rate of 7 sec per 10 Gy radiation. The irradiation procedure was executed in the gamma chamber (GC-6000) of RNARC, BCKV, West Bengal, India. Formal safety precautions, i.e., maintaining a safe distance of 10 m, using proper gloves and kits, closing the room during irradiation procedures, etc., were appropriately followed. A few irradiated seeds of each dose along with the untreated one (used as control; denoted by the irradiation dose of 0 Gy above) were germinated following the petri-plate method. Each filter paper (size 9), soaked with double-distilled water, was placed on twenty-one petri plates, properly marked with the dosage. Seeds of each dose were grown on those specifically marked Petri plates and incubated at 25±2 °C in the laboratory. Ten seeds of each irradiation dose and control were grown on Petri plates with three replications. For each dose, germination percentage (%), various seedling parameters *viz*. shoot length (cm), root length (cm), seedling height (cm), seedling vigor index, etc., were recorded for 7 and 14 days, respectively.

*2.2. Increase in M_1_ generation*

Excluding the GR_50_ value determining experiment, the rest of the seeds irradiated with six doses of gamma rays (100, 150, 200, 250, 300, and 350 Gy) along with the control (untreated seeds) were sown in the field. A few pollens were collected from the floral buds for the following experiment during the preflowering stage. From the rest, a large number of M_1_ populations were developed from those seeds. A large number of pods as well as seeds were collected from individual plants from the population for raising the next generation, *i.e.,* M_2_ population.

*2.3. Pollen fertility study*

Pollens were collected from the floral buds of irradiated seed-grown *in vivo* M1 plants along with controls. After collection, the pollens of each dose and control were treated with 1% (w/v) iodide-potassium iodide (IKI) [27] solution for a few seconds, after which they were minutely observed under the 40X objective lens of a compound light microscope (Olympus OIC) in the laboratory, as mentioned earlier. The pollen fertility percentage was calculated by implementing the following formula:

$$Pollen fertility percentage =\frac{Number of fertile pollens in a given area}{Number of total pollens in that given area}\times100$$

Pollens of all doses and the control were observed under the microscope with three replications each. Pollens from control plants were compared with those of each dose to check whether there was any reduction in pollen fertility percentage.

*2.4. Identification of candidate mutants in the M2 generation*

Seeds obtained from the M_1_ generation were sown in the next generation for the purpose of growing the M_2_ population in the aforementioned field using a plant-to-progeny method. Row-to-row and plant-to-plant spacings of 30 cm and 15 cm, respectively, were maintained. Individual plants were selected across the field based on their chlorophyll content and numerous morphological characteristics to determine the mutagenic effects of the acute gamma irradiation dosages (0, 100, 150, 200, 250, 300, and 350 Gy) administered in the previous generation. After ten days of germination, those candidate mutants were appropriately tagged and recorded. Additionally, a dose-dependent normal plant population was also determined. The observed plant mutants were handled as per the relevant regulations and guidelines of IAEA.

*2.5. Statistical data analysis*

The experiment involving the calculation of the dose of growth reduction was organized in a completely randomized block design with seven levels of gamma irradiation (including control) and three treatments, where the radiation levels were randomly arranged. One-way analysis of variance (ANOVA) for all the parameters studied in the experiments was carried out at a 5% significance level to test whether the observed averages of the treatment levels were significantly different. Probit analysis [28] was executed to determine the GR_50_ value of all the parameters of interest. All statistical analyses were performed using the SPSS (version 20.0, SPSS Inc., Chicago, IL, USA) software package.

**3. Results**

*3.1. GR_50_ value*

In this study, the germination percentage was highest (100%) in the control seeds, whereas retardation in germination efficiency and germination percentage was found with increasing radiation dose (Figure 1 and 3a). Similarly, the control seedlings showed the best outcome concerning all other seedling parameters, *i.e.*, shoot length, root length, total seedling length, seedling vigor index, etc. (Table 1). The seedling vigor index was calculated according to the formula germination percentage × seedling length for each dose, which also provided a similar graph to seedling height in this study (Figure 3b, Table 1). Based on the total seedling length and root length, the GR_50_ value was measured, which was 217.2 Gy (Figure 2a and b).

**Figure 1.** Immediate effect of different doses of gamma irradiation (0, 100, 150, 200, 250, 300 and 350 Gy) on seedling parameters of lentil (*Lens culinaris* Medik.). (a)Growth status of seedlings after 7 days of experiment. (b)Growth status of seedlings after 14 days of experiment.

**Table 1.** Effect of different doses of gamma radiation on seedling parameters and pollen fertility status in lentils

| **Dose of radiation** | **Germination percentage** | | **Shoot length (cm)** | | | | | **Root length (cm)** | | | | | **Seedling length (cm)** | | | | | **Seedling vigor index** | | | | | **Pollen fertility percentage** | | |
| --- | --- | --- | --- | --- | --- | --- | --- | --- | --- | --- | --- | --- | --- | --- | --- | --- | --- | --- | --- | --- | --- | --- | --- | --- | --- |
|  | **7 DAS** | **14 DAS** | | | **7 DAS** | | **14 DAS** | | | **7 DAS** | | **14 DAS** | | | **7 DAS** | | **14 DAS** | | | **7 DAS** | | **14 DAS** | | |  |
| Control (0) | 100 | 100 | | 9.06±0.06 | | 13.6±0.21 | | | 3.17±0.32 | | 3.86±0.35 | | | 12.23±0.38 | | 16.47±0.14 | | | 1223.33±38.44 | | 1646.67±14.53 | | | 85.27±1.06 | |
| 100 | 97.67±0.33 | 97.67±0.33 | | 6.06±0.12 | | 10.63±0.71 | | | 1.76±0.3 | | 2.3±0.26 | | | 7.63±0.29 | | 12.77±1.03 | | | 745.67±30.83 | | 1247.57±104.98 | | | 69.5±1.85 | |
| 150 | 94.67±0.33 | 94.67±0.33 | | 4.6±0.15 | | 7.3±0.21 | | | 1.63±0.44 | | 1.76±0.67 | | | 6.33±0.38 | | 8.67±0.48 | | | 599.3±34.16 | | 820.13±42.57 | | | 59.43±1.29 | |
| 200 | 93±0.57 | 93±0.57 | | 3.6±0.05 | | 6.87±0.12 | | | 1.56±0.17 | | 1.63±0.44 | | | 5.1±0.05 | | 8.17±0.34 | | | 474.33±7.33 | | 759.73±35.5 | | | 52.16±1.64 | |
| 250 | 89.33±0.33 | 89.33±0.33 | | 2.73±0.23 | | 4.8±0.05 | | | 1.5±0.11 | | 1.56±0.08 | | | 4.5±0.05 | | 5.07±0.03 | | | 401.97±3.94 | | 452.63±4.09 | | | 45.2±1.39 | |
| 300 | 86.67±0.67 | 86.67±0.67 | | 2±0.11 | | 2.96±0.06 | | | 1.46±0.26 | | 1.43±0.44 | | | 3.47±0.17 | | 4.4±0.37 | | | 300.67±17.58 | | 381.4±33.08 | | | 36.76±2.11 | |
| 350 | 83.67±0.67 | 83.67±0.67 | | 0.96±0.03 | | 1.97±0.03 | | | 0.66±0.08 | | 1.3±0.35 | | | 1.63±0.12 | | 3.27±0.38 | | | 136.5±9.07 | | 272.8±30.24 | | | 28.1±1.53 | |

^1^ Tables may have a footer.

**Figure 2.** Dose response curve of (a) seedling length. (b) root length.

**Figure 3.** Graphical representation of effect of different doses of acute gamma irradiation (0, 100, 150, 200, 250, 300, and 350 Gy) on (a)gemination percentage at 7 and 14 days after experiment, respectively. (b) seedling vigor index at 7 and 14 days after experiment, respectively

*3.2. Pollen fertility*

Similar to the last experiment, the floral buds of the control seedlings were found to carry the maximum number of fertile pollens (almost 85%) (Figure 4a, Table 1). On the other hand, 350 Gy gamma-irradiated pollens showed the lowest fertility (27-29%) among all others (Figure 4g, Table 1). Notably, the pollens also exhibited the fertility percentage in descending order (Figure 4a-g) when placed with elevating doses of gamma radiation in the graph (Figure 4h).

**Figure 4.** Effect of gamma irradiation on fertility percentage of pollen of lentil (*Lens culinaris* Medik.) observed under 40X opjective lens of compound light microscope (Olympus OIC);the irradiation dosesare: (a) Control (0 Gy). (b) 100 Gy. (c) 150 Gy. (d) 200 Gy. (e) 250 Gy. (f) 300 Gy. (g) 350 Gy; (h) Graphical representation of the same.

*3.3. Frequency of candidate mutants in the M_2_ generation*

During the M_2_ generation, potential mutants (Figure 5) were thoroughly identified using agronomic and morphological parameters. Four distinct kinds of chlorophyll mutants, viz., albina (Figure 6b), xantha (Figure 6c), chlorina (Figure 6d), and xantha-viridis (Figure 6e), were identified (Table 2). Additionally, a few leaf morphological (Figure 6i-t, v, w) and plant morphological mutants (Figure 6h-j) were also detected (Table 2).

**Figure 5.** Diagram of development of chloro- and morpho-mutants in M_2_ generation.

**Figure 6**. Detection of chlorophyll and morphological mutants in M_2_ population. (a, g, k) Untreated plant, (b-e) Chlorophyll mutants [(b) Albina, (c) Xantha, (d) Chlorina, (e) Xantha-viridis], (f) Chimera, (h-j) Plant morphological mutants, (i-t) Leaf shape (leaf morphological) mutants, (u) Leaf arrangement in untreated plant, (v-w) Change in leaf arrangement in leaf morphological mutants

**Table 2: Frequency of candidate mutants obtained in the M_2_ generation**

| **Types of mutants** | | **Frequency of mutants obtained in specific doses of gamma radiation (%)** | | | | | | **Total frequency (%)** |
| --- | --- | --- | --- | --- | --- | --- | --- | --- |
|  |  | **100 Gy** | **150 Gy** | **200 Gy** | **250 Gy** | **300 Gy** | **350 Gy** |  |
| Chlorophyll mutants | Albina | - | - | - | 0.001 | 0.001 | - | 0.002 |
|  | Xantha | - | 0.002 | 0.007 | 0.01 | 0.013 | 0.008 | 0.031 |
|  | Chlorina | - | 0.005 | 0.017 | 0.025 | 0.033 | 0.011 | 0.091 |
|  | Xantha-viridis | - | 0.003 | 0.012 | 0.016 | 0.019 | - | 0.05 |
|  | Chimera | - | - | 0.001 | 0.002 | 0.004 | - | 0.007 |
| Leaf morphological mutants | Change in leaf shape | - | 2.43 | 5.57 | 9.63 | 13.43 | 7.69 | 38.75 |
|  | Change in leaf arrangement | - | 0.001 | 0.001 | 0.003 | 0.004 | - | 0.009 |
| Plant morphological mutants | | - | - | 0.007 | 0.008 | 0.011 | 0.003 | 0.029 |

4. Discussion

Prior to initiating experiments on colossal scale mutant breeding, LD_50_ and GR_50_ dosages are essential [29]. These GR_50_ and LD_50_ values are crucial because they result in a greater likelihood of acquiring desirable mutants [22]. According to Álvarez-Holguín, et al. [30], lower doses of gamma irradiation result in minor alterations to the genome, but higher doses result in undesired or deadly mutations, *i.e.,* the higher the doses, the greater the mortality. Therefore, the undesirable mutations obtained remain a way away from the expected conclusion [30]. Moreover, it is already well established in accordance with the norms of IAEA that gamma irradiation up to the dosage of 10 kGy had no deleterious effect on human health [31]. Therefore, seeds treated with gamma rays should be considered safe when used for consumption purposes.

Probit analysis, used in the present study, is extensively utilized in research involving binomial responses. Its primary application is in toxicological investigations, where it converts the sigmoidal dose‒response curve to a straight line that can then be easily evaluated using regression deploying either least squares or maximum likelihood. To simplify the complex percent affected vs. dose‒response relationship into a simple linear relationship of probit vs. dose‒response, 0 is defined as 0.0001, and 10 as 0.9999 and included a table to assist researchers in converting impact-percentage to probit, which is then plotted against the logarithm of dose. Moreover, this existing tool has the capability to evaluate LD_50_ or GR_50_ at a 95% confidence interval along with a chi-square test for checking the adequacy of fit [32].

The determined GR_50_ value in this research, through probit analysis, was 217.2 Gy. In general, GR_50_ is an effective method for quantifying a parameter's 50% decrease [23]. Because M_1_ plants are heterozygous at the genotypic level, the change caused by mutation may be seen in any allele located in any random locus, leading to reduced mutation manifestation [33]. A lower likelihood of detecting a recessive mutation at this stage and how a larger segregating mutant population may be used to harvest unique mutations in the future were elaborated in previous studies. Additionally, the GR_50_ study may be regarded as acceptable since a minimal percentage of deaths occurred compared to the LD_50_ study.

Pollen fertility research has also examined the tissue-damaging properties of gamma rays. Similar to seedling height, the greater the dosage of gamma radiation, the lower the pollen fertility %. This conclusion was already endorsed by a number of studies [34-39]. Kumar, et al. [35] demonstrated that when dried seeds of pea were irradiated, a 5 kR dosage of gamma rays induced 10.06 percent pollen sterility, while a 40 kR exposure generated 53.12 percent pollen sterility. Additionally, when presoaked seeds were utilized, they observed an increase in pollen sterility. Wani [39] treated lentil seeds from the Pant L-406 and Type-8 varieties with three different chemical mutagens (EMS, sodium azide, and hydrazine hydrate). Additionally, this experiment demonstrated a decrease in pollen fertility and increased dosages of each mutagen. Notably, for each dosage, the M_2_ generation had a higher fertility % than the M_1_ generation [39]. Additionally, Priyanka, et al. [36], Eswaramoorthy, et al. [34], and Vikhe and Nehul [38] all found comparable findings with the mutagens they employed in horse grams (*Macrotyloma uniflorum*), cowpea (*Vigna unguiculata* (L.) Walp. ), and mung bean (*Vigna radiata* (L.) Wilczek). On the other hand, Tamilzharasi, et al. [37] showed a reduced pollen fertility % in black gram (*Vigna mungo* (L.) Hepper) when combined higher doses of gamma rays and EMS were used rather than only gamma rays.

Albina, chlorina, xantha, and xantha-viridis chlorophyll mutants were obtained in the present investigation. Chlorina was the most prevalent, accounting for 0.091 percent of the total, followed by xantha-viridis (0.05 percent), xantha (0.031 percent), and albina (0.002 percent) (Table 2). Albina was detected only in plots with seeds that had been irradiated with 250 Gy and 300 Gy gamma rays. In the case of other chlorophyll mutants, the frequency increased up to 300 Gy but then decreased abruptly after 350 Gy gamma ray exposure. This might be due to the fatality associated with greater doses of this physical mutagen. Similarly, only plots with 200 Gy, 250 Gy, or 300 Gy sowed chimera at frequencies of 0.001%, 0.002%, and 0.004%, respectively (Table 2). The highest frequency was reported when morphological mutants with altered leaf shape were detected (38.75%). Nine distinct leaf forms were identified from this group (Figure 6i-t). Additionally, two different forms of leaf arrangement mutations (Figure 6v, w) and three other types of plant morphological mutants were detected in the field (Figure 6h-j). Furthermore, the plants treated with 300 Gy had the highest frequency of mutants, followed by the plants treated with 250 Gy.

The frequency of chlorophyll mutation is an excellent indicator of various mutagen dosages [40]. Additionally, chlorophyll abnormalities generated by irradiation may be utilized to locate accessible mutations in the irradiated population. Ionizing radiation has the potential to modify the photosynthetic complex [41] by decreasing the photosystem's efficiency [42]. A substantial dosage of gamma rays, up to 500 Gy, lowers the chlorophyll content by 80.91% and the grana and stroma thylakoid organization patterns [43]. Apart from those fundamental outcomes, stable mutants with good agronomic performances can directly be used as breeding materials for making novel genotypes [44, 45].

5. Conclusions

The current study examines the immediate impact of a wide range of acute gamma irradiation doses (0 Gy to 350 Gy) on lentil seed germination, seedling growth characteristics, and pollen fertility status in the absence of any other experimental manipulation. Additional results of the study showed that higher doses of acute gamma irradiation resulted in greater damage to seedling growth and pollen fertility than lower doses across all of the parameters studied. Following an extensive investigation into seedling characteristics, it was established that the GR_50_ value was 217.2 Gy. Given the probability of gaining a higher mutation frequency in the future generation, it is possible that this amount of gamma radiation would be considered beneficial. As a consequence, by conducting additional studies, it is possible to employ this specific dose to develop a unique variety in lentils. This research will pave the way for a lentil improvement program that will use mutant breeding as a cost-effective technique to boost yields and develop new genotypes to be released as a variety.

**Acknowledgment**: The research article is a part of Mr. Biswajit Pramanik for his Ph.D. research program, which was supervised by Dr. Sandip Debnath at Visva-Bharati University. Researchers also acknowledge the help received from the YSRA-BRNS project entitled “Physical mutation to improve P acquisition along with nodulation efficiency in lentils”, (No. 55/14/17/2020-BRNS/10360 Dated 10/12/2020) from the Department of Atomic Energy, BARC, Mumbai, India.

**Data availability**

All data generated or analyzed during this study are included in this article.

**Conflict of Interest**

The authors declare that they have no conflicts of interest.

**Funding Statement**

Not applicable.

References

[1] S. Sharma, M. Knox and T.N. Ellis, "AFLP analysis of the diversity and phylogeny of Lens and its comparison with RAPD analysis," *Theoretical and Applied Genetics*, vol. 93, no. 5, pp. 751-758, 1996.

[2] M.C. Saxena, "Plant morphology, anatomy and growth habit." In *The lentil: Botany, production and uses*, edited by W. Erskine, F.J. Muehlbauer, A. Sarker and B. Sharma, pp 34-46. Wallingford, UK: CABI Press, 2009.

[3] J. Duke, *Handbook of legumes of world economic importance.* USA: Springer Science & Business Media, 2012.

[4] A. Cokkizgin and M.J. Shtaya, "Lentil: Origin, cultivation techniques, utilization and advances in transformation," *Agricultural Science*, vol. 1, no. 1, pp. 55-62, 2013.

[5] R. Bhatty, "Composition and quality of lentil (*Lens culinaris* Medik): a review," *Canadian Institute of Food Science and Technology Journal*, vol. 21, no. 2, pp. 144-160, 1988.

[6] S. Debnath, A. Sarkar, K. Perveen, N.A. Bukhari, K.K. Kesari, A. Verma, N.R. Chakraborty and M. Tesema, "Principal component and path analysis for trait selection based on the assessment of diverse lentil populations developed by gamma-irradiated physical mutation," *BioMed Research International*, vol. 2022, pp. Article ID 9679181, 14 pages, 2022.

[7] V. Yankova and S. Sovkova-Bobcheva, "Studying of bean varieties (*Phaseolus vulgaris* L.) reaction to bean weevil infestation (Acanthoscelides obtectus Say)." In *Reports of bean improvement cooperative and national dry bean council research conference*, 144-145. Michigan State University, USA, 2009.

[8] R.A. Laskar, S. Khan, C.R. Deb, N. Tomlekova, M.R. Wani, A. Raina and R. Amin, "Lentil (*Lens culinaris* Medik.) diversity, cytogenetics and breeding." In *Advances in plant breeding strategies: legumes*, edited by J. Al-Khayri, S. Jain and D. Johnson, pp 319-369: Springer, Cham, 2019.

[9] M. Materne and D.L. McNeil, "Breeding methods and achievements." In *Lentil: an ancient crop for modern times*, edited by S.S. Yadav, D.L. McNeil and P.C. Stevenson, pp 241-253. Dordrecht, Netherlands: Springer, 2007.

[10] M.E. Ferguson, N. Maxted, M. Van Slageren and L.D. Robertson, "A reassessment of the taxonomy of *Lens Mill.* (Leguminosae, Papilionoideae, Vicieae)," *Botanical Journal of the Linnean Society*, vol. 133, no. 1, pp. 41-59, 2000.

[11] R. Amin, R.A. Laskar and S. Khan, "Assessment of genetic response and character association for yield and yield components in Lentil (*Lens culinaris* L.) population developed through chemical mutagenesis," *Cogent Food & Agriculture*, vol. 1, no. 1, pp. Article 1000715, 15 pages, 2015.

[12] J.F. Ali, M. Arain and N. Shaikh, "Genetic manipulation of lentil through induced mutations," *Pakistan Journal of Botany*, vol. 42, no. 5, pp. 3449-3455, 2010.

[13] Y. Du, Z. Feng, J. Wang, W. Jin, Z. Wang, T. Guo, Y. Chen, H. Feng, L. Yu and W. Li, "Frequency and spectrum of mutations induced by gamma rays revealed by phenotype screening and whole-genome resequencing in Arabidopsis thaliana," *International Journal of Molecular Sciences*, vol. 23, no. 2, pp. Article 654, 2022.

[14] G. Abbas, M.J. Asghar, M. Rizwan and F. Ahmad, "Harnessing positive variability in F2 and M2 populations of lentil," *Journal of Agriculture and Basic Sciences*, vol. 1, pp. 37-45, 2016.

[15] S. Akhtar, M. Ahsan, M.J. Asghar, G. Abbas, M. Rizwan and Z. Ahmad, "F1 seed mutagenesis: A novel technique of creating genetic variability in lentil," *Journal of Agriculture and Basic Sciences*, vol. 2, no. 2, pp. 45-51, 2017.

[16] M.A. Baghery, S.K. Kazemitabar and R.E. Kenari, "Effect of EMS on germination and survival of okra (*Abelmoschus esculentus* L.)," *Biharean Biologist*, vol. 10, no. 1, pp. 33-36, 2016.

[17] A.K. Gaur, I. Singh, S. Singh and K. Reddy, "Studies on effects of gamma ray doses on germination in pigeonpea [*Cajanus cajan* (L.) Millspaugh] under laboratory and field conditions," *International Journal of Chemical*, vol. 6, no. 4, pp. 1975-1977, 2018.

[18] S. Khursheed, K. Waheed, I.U. Haq and S. Khan, "Mutagenic damage by gamma rays and ethyl methanesulphonate in two cultivars of Faba bean," *International Journal of Botany Studies*, vol. 5, no. 5, pp. 50-54, 2020.

[19] R.A. Laskar and S. Khan, "Assessment on induced genetic variability and divergence in the mutagenized lentil populations of microsperma and macrosperma cultivars developed using physical and chemical mutagenesis," *PloS one*, vol. 12, no. 9, pp. e0184598, 2017.

[20] R. Pandit, B. Bhusal, R. Regmi, P. Neupane, K. Bhattarai, B. Maharjan, S. Acharya, K. Bigyan and M.R. Poudel, "Mutation breeding for crop improvement: a review," *Reviews in Food and Agriculture*, vol. 2, no. 1, pp. 31-35, 2021.

[21] M. Pharmawati, M.R. Defiani, L.P. Wrasiati and I.M.A.S. Wijaya, "Morphological changes of *Capsicum annuum* L. induced by ethyl methanesulfonate (EMS) at M2 generation," *Current Agriculture Research Journal*, vol. 6, no. 1, pp. 1-7, 2018.

[22] M.A. Wanga, H. Shimelis, L.N. Horn and F. Sarsu, "The effect of single and combined use of gamma radiation and ethylmethane sulfonate on early growth parameters in sorghum," *Plants*, vol. 9, no. 7, pp. Article 827, 2020.

[23] S. Layek, S. Pramanik, A. Das, A.K. Gupta, A. Bhunia and M.K. Pandit, "Effect of gamma radiation on seed germination and seedling growth of snake gourd (*Trichosanthes anguina* L.)," *South African Journal of Botany*, vol. 145, pp. 320-322, 2022.

[24] V.V. Kumari, P. Banerjee, R. Vijayan, R. Nath, K. Sengupta and M. Chandran, "Effects of micronutrient foliar spray on thermal indices, phenology and yield of lentil in new alluvial zone of West Bengal," *Journal of AgriSearch*, vol. 7, pp. 202-205, 2020.

[25] S. Mookherjee, J. Karforma, T.K. Pandit and T.S. Ghimiray, "Yield gap analysis ‘Infarmers’ participatory seed production plot of lentil (*Lens culinaris* Medic.) at old alluvial zone of West Bengal, India," *Journal of Pharmacognosy and Phytochemistry*, vol. 8, no. 2, pp. 449-451, 2019.

[26] S. Roy, F. Rahman, P. Pal and J. Basak, "Enhancing pulse productivity through agro-technologies under clustered frontline demonstration programme." 1-25. Bihar, Jharkhand and West Bengal, 2018.

[27] V. Jayashree, A. Muthuswamy, P. Jayamani and K. Kumar, "Interspecific hybridization and crossability studies of cultivated varieties of *Vigna mungo* L. Hepper with Vignamungo var silvestris," *Electronic Journal of Plant Breeding*, vol. 11, no. 01, pp. 271-275, 2020.

[28] D.J. Finney, *Probit analysis* Cambridge, UK: Cambridge University Press, 1971.

[29] V. Kumar, G. Vishwakarma, A. Chauhan, A. Shitre, B.K. Das, J. Nair, P. Surendran, H. Sparrow and A. Gupta, "Use of proton beam as a novel tool for mutations in rice," *BARC Newsletter*, vol. 366, pp. 5-9, 2018.

[30] A. Álvarez-Holguín, C.R. Morales-Nieto, C.H. Avendaño-Arrazate, R. Corrales-Lerma, F. Villarreal-Guerrero, E. Santellano-Estrada and Y. Gómez-Simuta, "Mean lethal dose (LD 50) and growth reduction (GR 50) due to gamma radiation in Wilman lovegrass (*Eragrostis superba*)," *Revista Mexicana de Ciencias Pecuarias*, vol. 10, no. 1, pp. 227-238, 2019.

[31] K. Dimov, "Effect of gamma irradiation on the primary and secondary products of lipid oxidation in raw chicken meat, stored under different temperatures and packaging–a meta-analysis," *Archiva Zootechnica*, vol. 25, no. 1, pp. 130-141, 2022.

[32] V. Kumar, O. Sheoran, S. Rani and K. Malik, "Development of a web-based tool for probit analysis to compute LC50/LD50/GR50 for its use in toxicology studies," *Journal of Applied and Natural Science*, vol. 12, no. 4, pp. 641-646, 2020.

[33] R. Roychowdhury and J. Tah, "Mutagenesis—A potential approach for crop improvement." In *Crop improvement*, edited by K. Hakeem, P. Ahmad and M. Ozturk, pp 149-187. Boston, USA: Springer, 2013.

[34] V. Eswaramoorthy, T. Kandasamy, K. Thiyagarajan, C. Vanniarajan and S. Jegadeesan, "Effectiveness and efficiency of electron beam in comparison with gamma rays and ethyl methane sulfonate mutagens in cowpea," *Applied Radiation and Isotopes*, vol. 171, pp. Article 109640, 2021.

[35] A. Kumar, A. Chaurasia, S. Marker, P. Shukla, P.K. Rai, P.K. Verma and B.M. Bara, "Effect of gamma radiation of macro mutations, effectiveness and efficiency under M2 generation in Pea (*Pisum sativum* L.)," *Annales of West University of Timisoara. Series of Biology*, vol. 19, no. 1, pp. 71-76, 2016.

[36] S. Priyanka, R. Sudhagar, C. Vanniarajan, K. Ganesamurthy and J. Souframanien, "Gamma rays induced morphological, flowering and palynological modifications in horse gram (*Macrotyloma uniflorum*)," *Journal of Environmental Biology*, vol. 42, no. 5, pp. 1363-1369, 2021.

[37] M. Tamilzharasi, D. Kumaresan, V. Thiruvengadam, J. Souframanian and P. Jayamani, "Assessment of mutagenic sensitivity in blackgram variety CO 6," *Electronic Journal of Plant Breeding*, vol. 12, no. 2, pp. 413-420, 2021.

[38] A.C. Vikhe and J.N. Nehul, "Mutagenic Efficiency and Effectiveness of EMS, SA and GR on *Vigna radiata* (L.) Wilczek," *World Journal of Current Scientific Research*, vol. 1, no. 1, pp. 76-80, 2021.

[39] M.R. Wani, "Comparative biological sensitivity and mutability of chemo-mutagens in lentil (*Lens culinaris* Medik)," *Legume Research*, vol. 44, pp. 26-30, 2019.

[40] S. Saha and A. Paul, "Radiation induced mutagen sensitivity and chlorophyll mutation frequency on sesame seeds," *Journal of Environmental Biology*, vol. 40, no. 2, pp. 252-257, 2019.

[41] H. Ali, Z. Ghori, S. Sheikh and A. Gul, "Effects of gamma radiation on crop production." In *Crop production and global environmental issues*, edited by K. Hakeem, pp 27-78: Springer, Cham, 2015.

[42] G. Angelini, P. Ragni, D. Esposito, P. Giardi, M. Pompili, R. Moscardelli and M. Giardi, "A device to study the effect of space radiation on photosynthetic organisms," *Physica Medica: PM: an International Journal Devoted to the Applications of Physics to Medicine and Biology: Official Journal of the Italian Association of Biomedical Physics (AIFB)*, vol. 17, pp. 267-268, 2001.

[43] S. Alikamanoglu and A. Sen, "Stimulation of growth and some biochemical parameters by magnetic field in wheat (*Triticum aestivum* L.) tissue cultures," *African Journal of Biotechnology*, vol. 10, no. 53, pp. 10957-10963, 2011.

[44] S. Debnath and S. Guha, "Breeding methods for quality improvement in horticultural crops." In *Value addition of horticultural crops: recent trends and future directions*, edited by A. Sharangi and S. Datta, pp 201-211. New Delhi. India: Springer, 2015.

[45] S. Satpathy, S. Debnath and A. Mishra, "Study on character association in *Lens culinaris* medik," *Electronic Journal of Plant Breeding*, vol. 12, no. 1, pp. 58-65, 2021.
